# Virologic and Immunologic Evidence Supporting an Association between HHV-6 and Hashimoto's Thyroiditis

**DOI:** 10.1371/journal.ppat.1002951

**Published:** 2012-10-04

**Authors:** Elisabetta Caselli, Maria Chiara Zatelli, Roberta Rizzo, Sabrina Benedetti, Debora Martorelli, Giorgio Trasforini, Enzo Cassai, Ettore C. degli Uberti, Dario Di Luca, Riccardo Dolcetti

**Affiliations:** 1 Section of Microbiology, Department of Experimental and Diagnostic Medicine, University of Ferrara, Ferrara, Italy; 2 Section of Endocrinology, Department of Biomedical Sciences and Advanced Therapies and LTTA, University of Ferrara, Ferrara, Italy; 3 Cancer Bio-Immunotherapy Unit, CRO – IRCCS, National Cancer Institute, Aviano, Italy; University of Pittsburgh, United States of America

## Abstract

Hashimoto's thyroiditis (HT) is the most common of all thyroid diseases and is characterized by abundant lymphocyte infiltrate and thyroid impairment, caused by various cell- and antibody-mediated immune processes. Viral infections have been suggested as possible environmental triggers, but conclusive data are not available. We analyzed the presence and transcriptional state of human herpesvirus 6 (HHV-6) in thyroid fine needle aspirates (FNA) and peripheral blood mononuclear cells (PBMCs) from 34 HT patients and 28 controls, showing that HHV-6 DNA prevalence (82% vs. 10%, p≤0.001) and viral load were significantly increased in FNA from HT patients, and thyrocytes from HT FNA displayed a 100-fold higher HHV-6 DNA load compared to infiltrating lymphocytes. In addition, while HHV-6 was strictly latent in positive samples from controls, a low grade acute infection was detected in HT samples. HHV-6 variant characterization was carried out in 10 HT FNA samples, determining that all specimens harbored HHV-6 Variant A.

The tropism of HHV-6 for thyroid cells was verified by infection of Nthy-ori3-1, a thyroid follicular epithelial cell line, showing that thyrocytes are permissive to HHV-6 replication, which induces *de novo* expression of HLA class II antigens. Furthermore, HHV-6-infected Nthy-ori3-1 cells become targets for NK-mediated killing, NK cells from HT patients show a significantly more efficient killing of HHV-6 infected thyroid cells than healthy controls, and HT patients have increased T-cell responses to HHV-6 U94 protein, associated to viral latency. These observations suggest a potential role for HHV-6 (possibly variant A) in the development or triggering of HT.

## Author Summary

Hashimoto's thyroiditis (HT) is a very common autoimmune disease of the thyroid. In addition to genetic background, several viruses, including herpesviruses, have been suggested to play a role as possible environmental triggers of disease, but conclusive data are still lacking. The anecdotal presence of human herpesvirus 6 (HHV-6) in HT specimens prompted us to study a possible association between HHV-6 and HT. Our analysis of fine needle thyroid aspirates and blood from HT patients and controls shows that HHV-6 prevalence and load are highly increased in HT patients. Furthermore, HT-derived thyrocytes harbor active virus, whereas HHV-6 is strictly latent in the few virus-positive controls. We also report that HHV-6 infects thyroid cells, inducing *de novo* expression of HLA-II surface antigens. Consequently, thyrocytes might behave as antigen presenting cells. Interestingly, immune cells from HT patients kill HHV-6-infected thyrocytes more efficiently than controls. Also, HT patients, but not controls, have specific T-cell responses to HHV-6 U94 protein. It is difficult to prove etiologic links between viral infections and diseases, especially in the case of a ubiquitous agent such as HHV -6. Nevertheless, our findings indicate that HHV-6 might contribute to HT development, and argue for a pathogenic association between HHV-6 and HT.

## Introduction

Hashimoto's thyroiditis (HT), or chronic lymphocytic thyroiditis, is a common autoimmune disease with unknown etiology and its prevalence has been increasing over the past 50 years [1], [2], [3]. Together with genetic factors, environmental factors are thought to be important in triggering autoimmune thyroid diseases (AITD), and viral infections have been suggested as possible environmental triggers [4], yet no conclusive evidence is available. Also herpesviruses have been suggested as potential cofactors, and have occasionally been detected in AITD [5], [6]. Thyroid cells infected with human cytomegalovirus were shown to act as antigen presenting cells and therefore might be involved in autoimmunity [7], patients with Graves' disease display a higher frequency of EBV-infected B cells secreting antibody to TSH-R [8], and AITD patients have elevated antibody titers against EBV antigens [9]. Human herpesvirus 6 (HHV-6) DNA has been detected in HT tissue specimens, but not in tissues from Graves' disease or multi nodular goiter [6].

HHV-6 infection is common and has a worldwide distribution [10]. Viral strains cluster in two variants: HHV-6A, with still unknown disease association, and HHV-6B, the etiologic agent of roseola (e*xanthem subitum*), a childhood benign febrile disease. HHV-6 *in vitro* replicates most efficiently in primary T-cells and in selected T-cell lines. However, the *in vivo* tropism of HHV-6 is considerably broader, including macrophages, endothelial cells, salivary glands, and brain [6], [11], [12]. After primary infection, HHV-6 establishes a latent infection and resides mainly in peripheral blood mononuclear cells (PBMCs) and in macrophages [11], [13]. During latency, HHV-6 expresses specific viral transcripts. In particular, expression of U94, in the absence of other viral lytic transcripts, is considered a molecular marker of viral latency [14], [15].

HHV-6 has been tentatively associated to several chronic autoimmune inflammatory processes [5], including Sjogren syndrome [16], [17], multiple sclerosis [18], [19], [20], rheumatoid arthritis and systemic lupus erythematosus [21], [22]. In addition, recent case reports suggested that HHV-6 infection might be related to the onset of autoimmune disorders, including purpura fulminans, severe autoimmune acquired protein S deficiency [23], autoimmune connective tissue diseases [24], and severe autoimmune hepatitis [25]. However, HHV-6 involvement in autoimmune diseases is still not supported by convincing data. In this report, we show that HHV-6 establishes a productive *in vivo* infection of thyroid cells from HT patients, that infected thyrocytes become a target for innate NK killing, and that HT patients have increased CD4+ and CD8+ T-cell responses to HHV-6 U94 protein. These findings strongly argue for a pathogenic association between HHV-6 and autoimmune HT.

## Results

### HHV-6 in clinical specimens

Thyroid fine needle aspirates (FNAs) derived from 34 HT patients and 28 patients with benign follicular epithelial lesions (controls) were analyzed for the presence of HHV-6 DNA by real time quantitative PCR (qPCR) specific for HHV-6 U94 and U42 genes.

All clinical samples were analyzed in double blind tests. In addition, when there was sufficient amount all samples were reanalyzed in a randomized and blinded fashion at a distant time from the first analysis, yielding superimposable data. All samples contained amplification-grade DNA, as shown by qPCR of RNase P, a cell reference gene that was also used to normalize viral loads to number of cells.

The results showed that HHV-6 was significantly more prevalent in HT FNAs (28/34, 82%) than in FNAs derived from controls (3/28, 10%)(p≤0.001) (Table 1). Furthermore, viral load was higher in HT specimens (mean 8.1×10^3^ copies/µg of cellular DNA, range 7.9×10^2^–4.2×10^4^ copies/µg DNA) than in the few controls which resulted positive for HHV-6 (mean 7.1×10^2^ copies/µg DNA, range 5.7×10^2^–8.2×10^2^ copies/µg DNA)(p≤0.05).

**Table 1:** Presence and transcriptional state of HHV-6 in specimens obtained from HT patients and controls.

Clinical Sample		Virus presence^1^	Virus load^1^	Virus Replication^2^	
				Latent	Active
**HT Patients**	**FNA**	28/34 (82%)	8.1×10^3^±1.5×10^3^	6/21 (28%)	15/21 (71%)
	**PBMC**	19/20 (95%)	1.2×10^4^±1.5×10^3^	18/18 (100%)	0/18 (0%)
**Controls**	**FNA**	3/28 (10%)	7.1×10^2^±1.2×10^2^	3/3 (100%)	0/3 (0%)
	**PBMC**	3/8 (37%)	3.7×10^2^±1.0×10^2^	3/8 (100%)	0/8 (0%)

1 Results obtained by qPCR analysis of total DNA extracted from fine needle aspiration biopsies (FNA) or peripheral blood mononuclear cells (PBMC), amplifying U94 and U42 genes. Virus presence is expressed as number of positive samples on the total number of tested samples (percentage of positivity in parenthesis). Virus load is expressed as the mean value of genome copy number in the positive samples ± SD. Differences were statistically significant in HT *vs* control FNAs (82% *vs* 10%, p≤0.001) and PBMCs (95% *vs* 37%, p≤0.01). Virus load was also significantly different in HT *vs* control FNAs (p≤0.05) and PBMC (p≤0.05).

2 HHV6 transcriptional state was analyzed by RT-qPCR performed on RNA extracted by the HHV6 DNA-positive specimens. Virus replication is defined as Latent (expression of U94 alone) or Active (expression of U94 and U42), and expressed as number of positive samples on the total number of tested samples.

In 10 specimens the amount of DNA was sufficient to characterize the viral variant by restriction enzyme pattern analysis of U31 nested PCR amplification product [26]. The results showed the presence of variant A in all tested specimens (data not shown).

**Figure 1:**
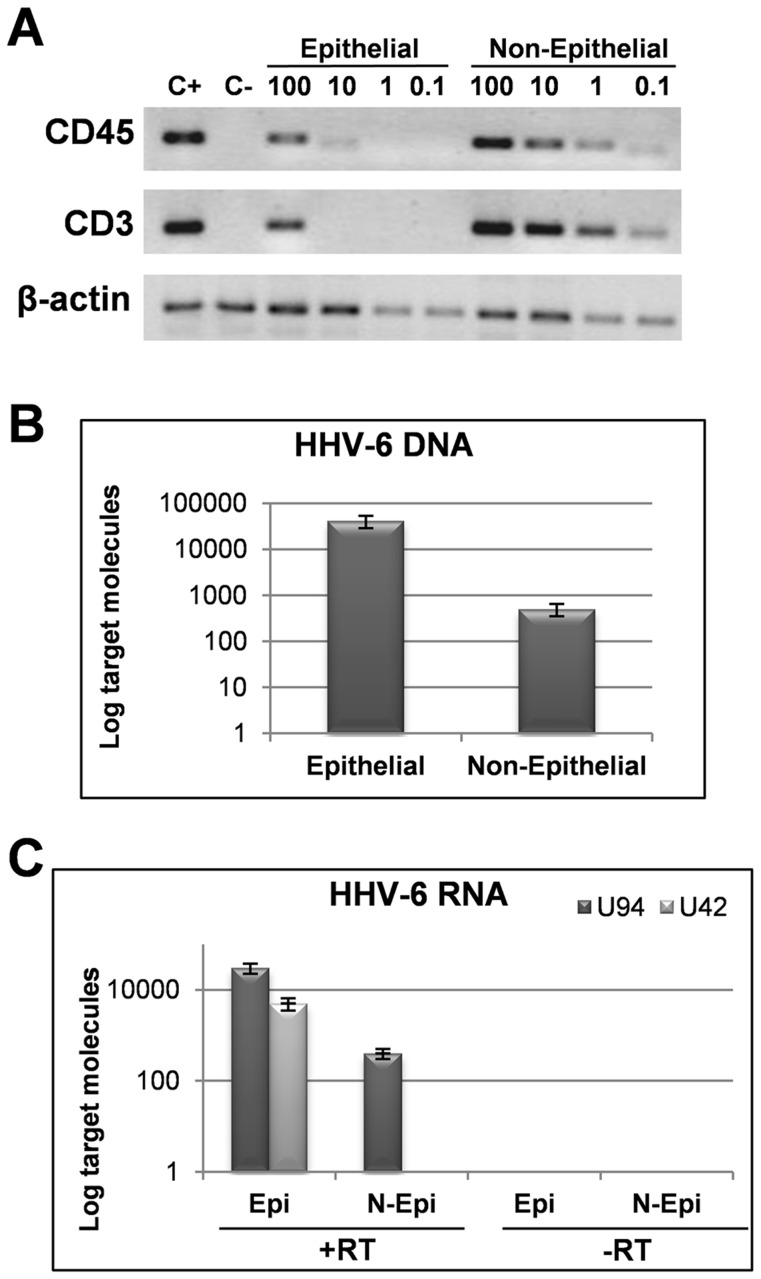
Presence and transcription of HHV-6 in epithelial and non-epithelial fractions of FNAs. (**A**) Cell fractions derived by immunomagnetic separation were characterized by RT-PCR specific for leukocyte transcripts (CD45, CD3), amplifying serial dilutions of cDNA template, corresponding to 100-10-1-0.1 ng of total RNA extracted from the individual FNA fractions. An amount of cDNA corresponding to 100 ng of RNA extracted from JJhan T cells or Nthy-ori3-1 thyroid cells was used respectively as positive (C+) or negative (C−) control. Amplified products were visualized on ethidium bromide stained 2% agarose gels. (**B**) HHV-6 DNA was searched by real time qPCR specific for U94 gene. Results are expressed in log scale and represent the mean copy number ± SD referred to duplicates of 2 independent assays averaged from two patients. Differences in virus load were statistically significant (p≤0.001). (**C**) HHV-6 transcription was analyzed by RT-qPCR for U42 and U94 transcripts. Results obtained in the absence of RT are also shown (RT−). Results are expressed in log scale and represent the mean copy number ± SD referred to duplicates of 2 independent assays. Transcription of U42 was significantly different (p≤0.001).

Due to the lymphotropic nature of HHV-6 and to the presence of significant lymphocyte infiltrates in thyroids of HT patients, we assessed whether HHV-6 was harbored in thyroid epithelial cells or rather in infiltrating lymphocytes. To address this question, FNA specimens from two additional HT patients were separated into epithelial and non-epithelial fractions by immunomagnetic selection with EpCAM (Ber-EP4) antibodies, and then analyzed for HHV-6 presence. Effective separation of fractions was checked by RT-PCR amplification of specific leukocytes transcripts (CD45, CD3), showing efficient depletion of lymphoid cells in the epithelial-enriched fraction (Figure 1A). The analysis of viral presence showed that HHV-6 was mainly harbored in the enriched epithelial fraction (i.e. thyroid cells), than in non-epithelial cells (i.e. lymphocytes), as shown by the observation that virus load was 100-fold higher in epithelial (mean value 4.1×10^4^ copies/µg DNA) than in non-epithelial (mean value 5×10^2^ copies/µg DNA) fraction (Figure 1B).

Since HHV-6 DNA was detected with high frequency in HT thyroids, we determined whether the virus was sustaining active or latent infection. To this purpose, we analyzed viral transcription in positive FNAs, by specific qPCR after retrotranscription (RT-qPCR) for U42 (indicative of productive replication) and U94 (expressed in both productive and latent phase). Therefore, the detection of U94 transcript in the absence of other viral mRNAs indicates latent infection. The analysis of the HHV-6 replicative status was performed in 21 out of the 28 HT patients that resulted positive for HHV-6. The analysis could not be performed in 7 specimens, since there was not enough material left for RNA studies. The results showed that 71% (15/21) of HT FNAs harbored transcriptionally active virus, whereas HHV-6 was strictly latent in the few positive controls, as shown by the detection of only U94 mRNA (Table 1). In addition, in the two samples which were separated into epithelial and non-epithelial fractions, lytic HHV-6 U42 transcripts were detected only in the epithelial-enriched fraction, while the lymphocyte fraction showed the presence of only latent U94 transcripts (Figure 1C), thus confirming that virus replication in thyrocytes and not in infiltrating lymphocytes.

HHV-6 establishes latent infection in peripheral T lymphocytes from the general population, thus HHV-6 presence and transcriptional state were also analyzed in peripheral blood mononuclear cells (PBMC) derived from HT patient (n = 20) and control (n = 8) groups. Viral DNA was detected in 19/20 (95%) HT patients and in 3/8 (37%) controls (Table 1). However, although viral loads were higher in HT patients (mean viral load of 1.2×10^4^ copies/µg DNA, range 7.5×10^2^–3.9×10^4^) compared to controls (mean viral load of 3.7×10^2^ copies/µg DNA, range 2.8×10^2^–4.9×10^2^)(p≤0.05), HHV-6 was exclusively latent in all PBMC samples, as revealed by the presence of U94 mRNA in the absence of U42 transcripts. A similar situation was found in healthy blood donors, where 6/20 (30%) collected PBMCs were found positive for HHV-6 presence (mean viral load of 3.6×10^2^ copies/µg DNA, range 1.2×10^2^–5.1×10^2^), and none of them harbored active virus, as revealed by the detection of only U94 transcript.

### HHV-6 infection of thyroid cells

The tropism of HHV-6 for thyroid cells has not been described before. Therefore, to assess whether thyrocytes are permissive to HHV-6, we performed *in vitro* infection of thyroid Nthy-ori3-1 with a HHV-6 cell-free inoculum, at a m.o.i. of 10 genome equivalents per cell. Viral replication was analyzed from 0 to 28 days post infection (d.p.i.), evaluating virus presence, transcription and antigen expression in infected cells, respectively by PCR, qPCR, RT-PCR and IFA.

**Figure 2:**
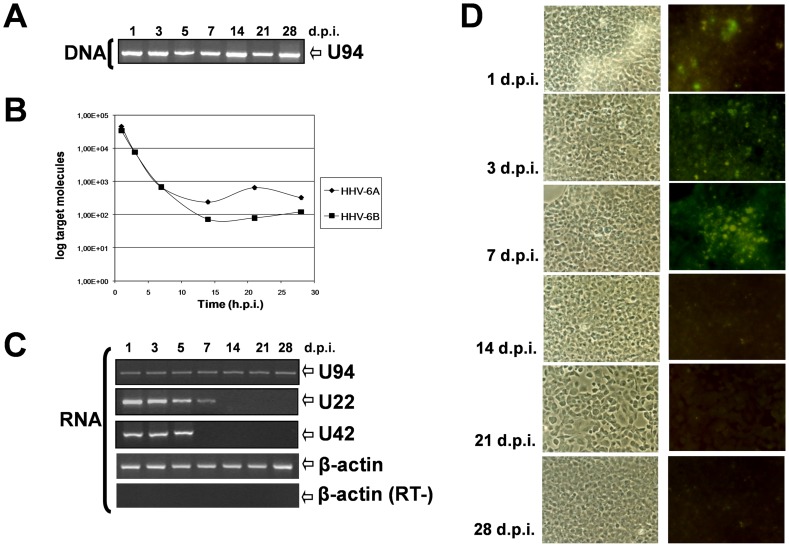
Replication of HHV-6 in thyroid cells. Nthy-ori3-1 cells were infected with HHV-6A or HHV-6B cell-free inocula, and cell pellets and supernatants were collected at the indicated days post infection (d.p.i.). HHV-6 DNA was searched by PCR (**A**) and quantified by real time qPCR (**B**). Results are shown as mean ± SD referred to duplicate samples of 3 independent experiments. HHV-6 transcription was analyzed by single round and nested RT-PCR (**C**), using total RNA extracted from infected cells at the indicated time points. RNA was retrotranscribed and amplified with primers specific for U22 (late gene), U42 (IE gene) and U94 (latency-associated gene). Amplification of β-actin is shown as positive control. Amplification of β-actin in the absence of RT is also shown. Virus antigen expression was analyzed by IFA (**D**), using a mouse mAb directed against HHV-6 gp116 late antigen. Images were taken in bright field (*left panels*) or blue light (*right panels*) with a UV light microscope (Nikon Eclipse TE2000S) equipped with a digital camera. Original magnification 100×. Data are shown for HHV-6A, but similar results were obtained also with variant B.

As shown in Figure 2A, HHV-6 DNA was present at all times p.i.. Virus load decreased from about 5×10^4^ copies/µg DNA at 1 d.p.i. (corresponding to 1 viral genome in 3 cells) to 5×10^2^ copies/µg DNA (1 viral genome in 300 cells) at 14 and 28 d.p.i., when the experiment was discontinued, with no substantial difference between HHV-6 variants (Figure 2B). Productive infection lasted for the first 7 d.p.i., as shown by the presence of U42 (immediate-early) and U22 (late) lytic transcripts. At later time points, only a strictly latent HHV-6 infection was observed, as shown by disappearance of lytic transcripts and the persistence of only U94 mRNA (Figure 2C). It is relevant to mention that U94 transcripts in lytically infected cells are expressed at lower levels relative to HHV-6 lytic genes, being approximately 100-fold less abundant than the transcripts of U42 gene, as previously reported by us [27] and in agreement with the original description by Rapp et al. [28]. Therefore the detection of only U94 mRNA, in the absence of lytic transcripts, is indicative of HHV-6 latency [14]. Likewise, the late viral antigen gp116 was detected for the first week p.i., and disappeared concomitantly to the establishment of latency (Figure 2D).

### HHV-6 up-regulates HLA class II antigens expression in thyrocytes

HLA class II antigens are not expressed by normal thyrocytes, but their expression can be induced by different stimuli, including IFN-γ [15] and virus infection [7]. Surface expression of HLA-II on thyroid follicular cells might induce an APC behavior, promoting the autoimmune responses underlying HT development. Thus we analyzed whether HHV-6 lytic or latent infection might induce HLA expression in thyroid cells. Nthy-ori3-1 cells were infected with HHV-6 and analyzed by flow cytometry at 24, 48, and 72 hours p.i.. In parallel, cells were also infected with HHV-7, as a control, or transfected with a U94-expressing plasmid, encoding the full length U94 gene, which is unique to HHV-6 and absent in the closely related HHV-7.

**Figure 3:**
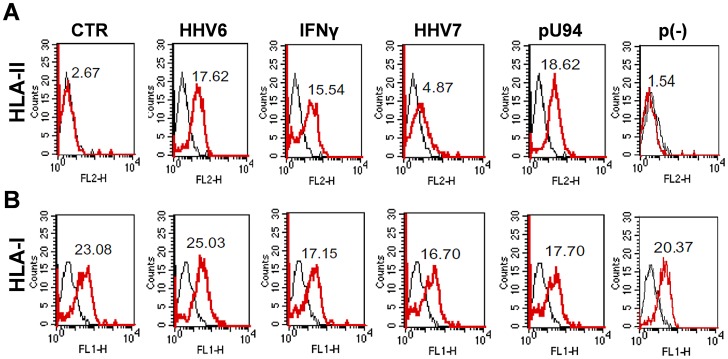
HLA-DR expression in HHV-6 infected thyroid cells. Nthy-ori3-1 cells were infected with HHV-6 and analyzed for HLA class II (**A**) or class I (**B**) expression. Cells were also infected with HHV-7, or transfected with U94 expression plasmid (pU94) or with the empty vector (p(−)). Treatment with IFN-γ was used as a positive control. Cells were collected at 24, 48, and 72 hours post infection or transfection, and analyzed by flow cytometry. Results refer to 72 hours post infection/transfection. Results are expressed as MFI (mean fluorescence intensity). (**A**): black lines represent isotype control; red lines represent labeled cells after treated with the different stimuli. (**B**): black lines represent isotype control; red lines represent cells treated with the different stimuli.

The results showed that HHV-6 infection induced cell-surface expression of DR antigenic determinants at a level comparable to that of IFN-γ treatment (17.62 and 15.54 MFI, respectively) (Figure 3A). By contrast, induction of HLA-II was not detected in HHV-7-infected cells (4.87 MFI), suggesting that the phenomenon is specific for HHV-6. HLA-II induction was instead observed in U94-transfected thyroid cells (18.62 MFI), whereas it was not detected in control cells receiving the same plasmid with no insert, suggesting that it might be related to the expression of this specific HHV-6 U94 gene. By contrast, no change was observed in HLA class I expression, neither in HHV-6 or U94 treated, nor in HHV-7 infected cells (25.03, 16.7, 17.7 MFI respectively, compared to the control 23.08 MFI value) (Figure 3B). These findings are consistent with the possibility that HHV-6 infection may induce thyrocytes to present immunogenic epitopes to CD4+ T cells through HLA-class II up-regulation.

### HHV-6-specific T cell responses in HT patients

**Figure 4:**
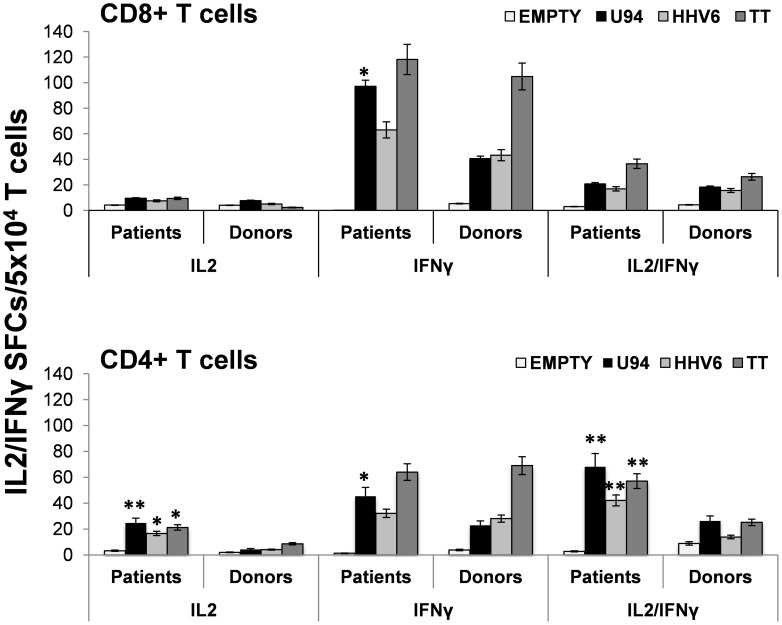
HHV-6-specific T cell responses in HT patients. Spontaneous T-cell responses to HHV6 lysate and the U94 protein was analyzed by IL2/IFN-γ dual color ELISPOT assay in 20 HT patients harboring active HHV-6 in their thyroid, and 20 healthy donors. Unloaded (EMPTY) and tetanus toxoid (TT) loaded monocytes were used respectively as negative and positive controls. The results of responses elicited in purified CD8+ (*Upper panel*) and CD4+ (*Lower panel*) T cells are shown. Responding cells were quantified as spot-forming-cells (SFCs), evaluated either on 50,000 CD8+ or CD4+ T cells. Data represent means ± SD of specimens, each tested in triplicate. The asterisks indicate that the mean number of T cells from HT patients responding to a specific antigen is significantly higher than that of T cells from healthy donors stimulated with the same antigen (_*_ = p≤0.05; _**_ = p≤0.001).

Due to the high prevalence of HHV-6 active infection in HT patients, we searched for the presence of HHV-6-specific T cell responses in those subjects, using a dual color ELISPOT assay. Assays were performed on CD8+ and CD4+T cells isolated from PBMCs derived from 20 HT patients harboring active HHV-6 in their thyroid, and 20 healthy blood donors, used as controls. T-cell responses were enumerated and characterized for the type of cytokine produced, using either total virus lysate or purified recombinant HHV-6 U94 protein as antigens. The results showed that HT patients carried higher numbers of virus-specific circulating CD8+ and CD4+ T cells, particularly those recognizing the U94 protein (Figure 4). Characterization of cytokine-secreting cells in response to HHV-6 lysate and U94 protein disclosed relevant differences between HT patients and donors. HT patients showed that enhanced virus-specific CD8+ T cell responses were limited to IFN-γ-producing cells to U94 (Figure 4, *Upper panel*). With regard to CD4+ T cells, HT patients showed significantly higher responses of IL-2 and IFN-γ/IL-2 secreting effectors to HHV-6 lysate and U94 protein, and also to control tetanus toxoid, consistently with an underlying over-activation of this compartment (Figure 4, *Lower panel*). Notably, however, patients displayed a selective and significant increase in CD4+ T cells secreting IFN-γ only in response to U94 protein. (Figure 4, *Lower panel*). These findings indicate that HT patients have significantly higher numbers of circulating HHV-6-specific CD4+ and CD8+ T cells, mainly secreting only IFN-γ and directed to the U94 protein.

### NK cell killing of HHV-6 infected thyrocytes

**Figure 5:**
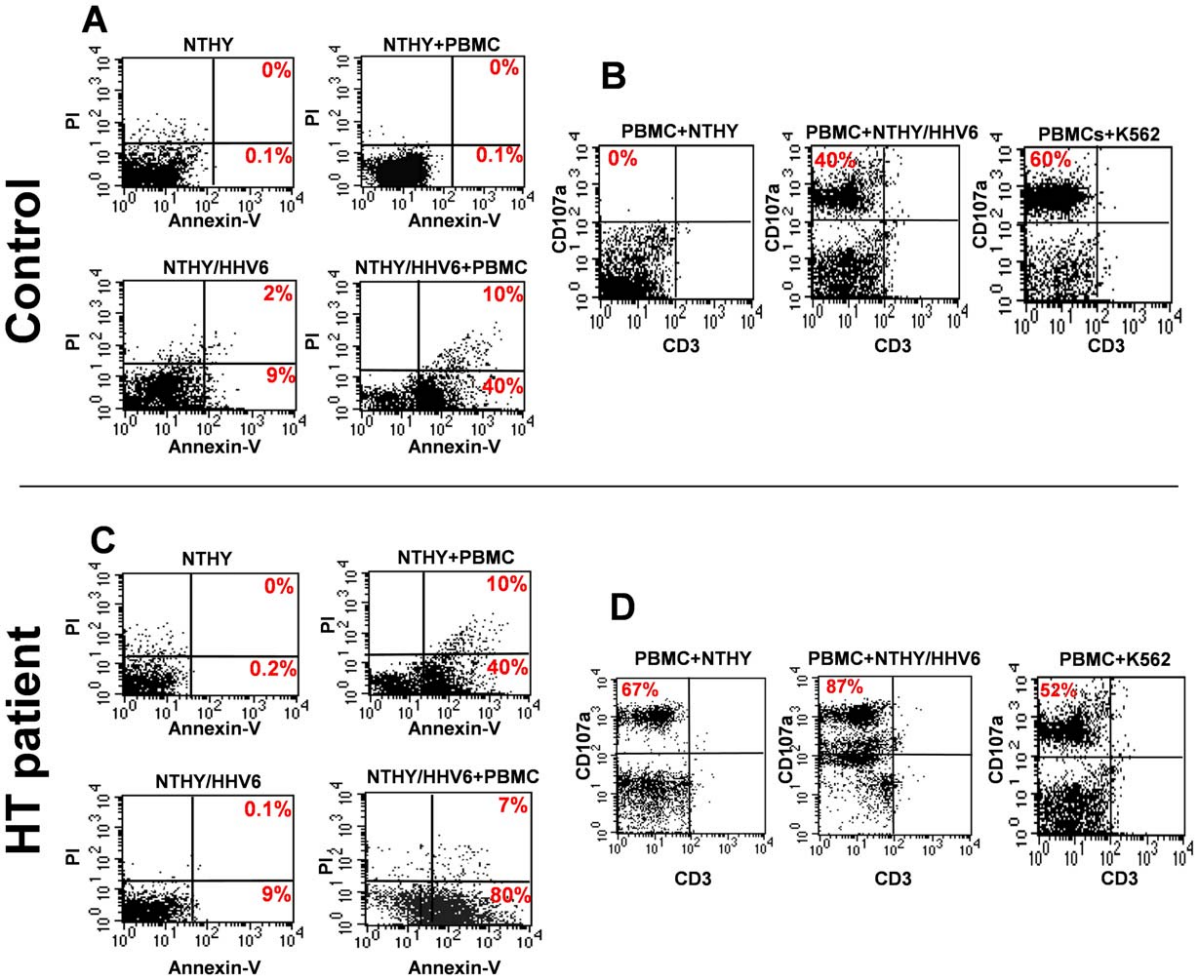
NK killing of infected thyrocytes. Cytotoxic activity associated with NK cell activation was analyzed in PBMCs from 3 controls and 3 HT patients. Nthy-ori3-1 cells at optimal density were infected with HHV-6 and cocultured with freshly isolated PBMCs at 48 hours post infection. After 4 hours of coculture, induction of apoptosis was evaluated by flow cytometry after staining with FITC-labeled Annexin-V, and cell viability was assessed by Propidium Iodide (**A, C**). In parallel, degranulation was assessed by staining with PE-Cy5-conjugated anti-CD107a mAb and gating on CD3−/CD56+ NK cells (**B, D**). NK degranulation *vs* K562 is also shown, as a positive control for standard cytotoxic assay. Results were obtained in triplicate samples. (**A, B**) Control subject, respectively induction of apoptosis (*left*) and degranulation (*right*) panels. (**C, D**) HT patients, respectively induction of apoptosis (*left*) and degranulation (*right*) panels. Results are representative for three independent experiments performed on PBMC from different HT and control subjects.

To ascertain whether HHV-6 infection increases the susceptibility of thyroid cells to destruction by innate immune cells, a specific *in vitro* cytotoxicity assay was used. Innate responses against HHV-6-infected Nthy-ori3-1 thyrocytes were investigated in PBMCs from 3 HT patients, previously shown to harbor active HHV-6 in their thyroids, and 3 healthy controls. Thyroid cells were infected with HHV-6 and used as target cells for the killing assay at 24, 48 and 72 h.p.i., using an effector∶target (E∶T) ratio of 2∶1. Co-cultures were incubated for 4 hours, a time chosen to detect mainly NK activation. Figure 5 illustrates the results obtained in one HT patient and one control, which are representative of all the subjects analyzed. Cell mortality associated with virus infection *per se* was very low (necrosis<2%)(Figure 5A, C), but significant apoptosis occurred in infected thyrocytes exposed to PBMCs from healthy donors (42±6%, average of three controls ± SD) (Figure 5A). Cytotoxic activity was associated with NK cell activation, as demonstrated by CD107a exposure on CD3−/CD56+ cells (Figure 5B). Notably, PBMCs from HT patients showed enhanced cytotoxic activity, both when cultured with uninfected (40±5% *vs* 0.1±0.08% in controls) and with HHV-6 infected thyrocytes (80±7% *vs* 40±3% in controls) (Figure 5C). Moreover, NK cells of HT patients displayed high levels of basal degranulation, even when cultured with uninfected thyrocytes (67±6% *vs* 0%) (Figure 5D). In parallel, CD107a expression by NK cells of HT patients increased substantially in the presence of HHV-6 infected cells, with more than 90% (91±4%) of CD3−/CD56+ NK cells activated. By contrast, degranulation of NK cells in a standard cytotoxic assay *vs* K562 showed similar levels in HT patients and controls, suggesting that the anti-HHV-6 NK activation observed in HT patients was not due to a general increase of NK cells reactivity (Figure 5B, D). However, due to the high basal level of apoptosis induced by HT NK cells on uninfected thyrocytes, an alternative explanation might be that HHV-6 infection sensitizes these cells to apoptosis-inducing effects.

## Discussion

Viral infections have been frequently cited as important environmental factors implicated in AITD [29], [30], but no specific virus has yet been conclusively associated to the disease. In particular, herpesviruses have been implicated, with conflicting evidence. Case reports suggested a potential association between herpesvirus infection and AITD [6], [9], [31], [32] but when thyroid FNAB specimens were analyzed no EBV, CMV or HSV-1 DNA was detected [33]. A recent study analyzed the presence of herpesvirus DNA in post-operative thyroid specimens from tissue blocks [6], and HHV-6 was detected by single round PCR in 2 out of 15 (13.3%) HT tissue specimens, whereas no HHV-6 DNA was isolated in Grave's Disease or Multi Nodular Goiter tissues.

In our report, employing a sensitive real time qPCR, 82% of FNAs from HT patients were found positive for HHV-6 DNA, whereas only 10% of control FNAs (derived from patients with benign thyroid lesions) harbored the virus. Furthermore, the majority of HT specimens harbored viral loads almost 2 logs higher compared to those of the few control thyroid tissues which resulted positive (p≤0.001). Interestingly, variant analysis performed in 10 HT samples showed that all samples harbored HHV-6A, suggesting that HT might be specifically associated to this variant.

Also the pattern of infection established by HHV-6 in HT patients is substantially different than that observed in healthy individuals, being characterized by: i) higher viral frequency and load in HT biopsies compared to controls; ii) active HHV-6 transcription in HT thyrocytes, consistent with virus replication, compared to latent infection in the few HHV-6-infected control thyroids; iii) presence of HHV-6 infection mainly in thyrocytes, rather than in lymphocytes infiltrating the lesion; iv) increased prevalence of latent HHV-6 infection in PBMCs.

These findings are consistent with the possibility that the thyroid of HT patients may constitute a site of active HHV-6 infection/replication. Considering that HT patients have increased HHV-6 prevalence in their PBMCs, where the virus is strictly latent, still unidentified microenvironmental factors are probably required to allow HT thyrocytes to be infected by HHV-6. Permissiveness to HHV-6 infection of thyrocytes was confirmed by our *in vitro* experiments showing that Nthy-ori3-1 cells support HHV-6 replication and antigen expression for approximately one week. Subsequently, viral latency is established, with a pattern similar to that recently described in endothelial cells [27]. Therefore, HHV-6 is capable of long-term persistence in thyrocytes, a relevant pre-requisite to exert possible pathogenic effects locally. Here we provide evidence indicating that HHV-6 may induce a *de novo* expression of HLA class II molecules in thyrocytes, which may thus behave as functional antigen presenting cells for CD4+ T lymphocytes. A similar observation has already been reported for human cytomegalovirus infection of thyroid cells [7], [34]. Intriguingly, enhanced HHV-6-specific T cell responses were observed in all HT patients, with a marked increase in the number of CD4+ T lymphocytes recognizing HHV-6 antigens, particularly the subset of polyfunctional CD4+ T cells secreting both IFN-γ and IL-2. These findings are consistent with an abnormal, probably persistent, immune response to HHV-6 antigens in HT patients, possibly favored by the local up-regulation of HLA class II molecules on thyrocytes induced by HHV-6 infection. These HHV-6-specific responses are likely embedded in a context of global over-activation of the CD4+ T cell compartment, as suggested by the increased responses of CD4+ T cells producing IL-2 or IFN-γ/IL-2 to the TT antigen. Nevertheless, HT patients showed significantly higher numbers of CD4+ and CD8+ T cells secreting IFN-γ only in response to the U94 antigen, suggesting a possible role of these effectors in mediating the killing of U94-expressing thyrocytes.

The finding that the HHV6 U94 antigen elicited higher responses than the whole HHV6 lysate could be explained by the fact that HHV-6 infection is ubiquitous and highly prevalent, with almost 100% individuals having memory T cells recognizing lytic viral antigens [35], [36], [37]. Instead, high expression of the regulatory, non-constitutive U94 protein of the virus and the consequent development of a specific immune response against U94, seems to be limited to specific pathologic conditions, as previously described also in multiple sclerosis patients, likely related to multiple virus reactivations [38]. Interestingly, autoimmune thyroid disease is significantly frequent in multiple sclerosis patients [39], [40], and it could be hypothesized that HHV-6 replication in HT patients might potentially be induced by autoimmune inflammation, as has been suggested for multiple sclerosis [41], [42]. Therefore, the occurrence of selective U94-specific CD4+ and CD8+ T cell responses in HT patients, suggests a specific role of this viral product as a potential trigger of autoimmunity. Alternatively, HT patients might experience variations in U94 production, or frequent switches between latency and active replication, leading to an increased sensitization to this viral antigen.

In addition to abnormal HHV-6-specific T cell responses, innate immunity triggered by HHV-6 may also contribute to HT development. In fact, PBMCs from HT patients showed a markedly enhanced cytotoxic activity to HHV-6-infected thyrocytes compared to control PBMCs derived from healthy donors. These findings, together with the observation that NK cells of HT patients show high levels of basal degranulation even when cultured with uninfected thyrocytes, suggest that these patients might suffer from an inherent NK cell alteration.

However, further studies are required to fully elucidate this association and the mechanisms underlying the possible role of HHV-6 as a trigger of HT. Indeed, there are several potential mechanisms by which HHV-6 might induce autoimmune responses. Viral infections might trigger autoimmunity by exposing high amounts of normally sequestered cell antigens, through lysis of infected cells. Another potential trigger is represented by molecular mimicry, with the synthesis of viral proteins that resemble cellular molecules, as a mechanism of immune escape. The virus could also induce aberrant expression of histocompatibility molecules thereby promoting the presentation of auto-antigens. Notably, HHV-6 has the ability to trigger all the above mentioned mechanisms and in the recent years, several reports have suggested a potential role of HHV-6 in autoimmunity [21], [24], [43], [44], [45], [46], [47]. Overall, our study indicates that HHV-6 infection might be an important factor in HT development.

## Materials and Methods

### Clinical samples

The samples were obtained as part of routine clinical work from patients undergoing fine needle aspiration biopsy (FNAB) for diagnostic purposes, and were used after receiving approval from the Local Ethical Committee of the University of Ferrara and S. Anna Hospital of Ferrara. The patients provided written informed consent for both FNAB procedure (which is part of the clinical practice) and for biomolecular analyses, to which purpose the samples were anonymized.

A total of 62 subjects participated in the study. None of them had other concomitant diseases or was taking drugs that could possibly affect thyroid function. All patients and controls were euthyroid at the moment of biopsy. Serum TSH, free T4, thyroperoxidase antibodies (TPO Ab; normal value<35 IU/ml) and thyroglobulin antibodies (Tg Ab; normal value<115 IU/ml) were measured in all patients by using an immuno-electrochemiluminescence technique (Modular E 170, Roche Diagnostics GmbH). All patients underwent ultrasound guided thyroid fine needle aspiration, as previously described [48]. Fine needle thyroid aspirates (FNAs) were used for both cytology and molecular analysis. The diagnosis of HT was based on the criteria described by Kini [49]. On the basis of these characteristics, 34 FNAs were considered as consistent with HT. The other 28 samples were FNAs derived from the normal tissue surrounding thyroid nodules in patients with hyperplastic follicular lesions (eg. Multi Nodular Goiter, MNG), and were considered as controls. The 34 HT patients included 7 males and 27 females, with a mean age of 49.8±2.5 years (range 32–75 years), with TPO Ab>35 IU/ml (mean value = 1312 IU/ml, range 146–8229 IU/ml), and Tg Ab>115 IU/ml (mean value = 751 IU/ml, range 280–3500 IU/ml). The 28 FNA control patients included 10 males and 18 females with a mean age of 53.3±5.6 years (range 33–88 years) (there was no statistically significant difference between the two groups), and showed TPO Ab<35 IU/ml (mean value = 9 IU/ml, range 7–11 IU/ml), and Tg Ab<115 IU/ml (mean value = 12 IU/ml, range 10–16 IU/ml).

In two HT cases, FNA amount allowed isolation of epithelial and lymphoid cells by immunomagnetic beads coated with an anti-EpCAM (Ber-EP4) antibody (CELLection Epithelial Enrich (Dynal AS, Oslo, Norway), following manufacturer instructions. Effective separation of fractions was checked by semiquantitative PCR amplification of specific leukocytes (CD45, CD3) transcripts.

Peripheral blood mononuclear cells (PBMCs) were isolated by Ficoll-Hypaque gradients. Aliquots of 10^6^ PBMCs were stored −80°C for DNA and RNA analyses, whereas aliquots of 10^7^ PBMCs were stored viably for ELISPOT analyses.

### Cell cultures, transfection and virus infection

Nthy-ori3-1 cells, a thyroid follicular epithelial cell line [50], were maintained in RPMI medium with 10% FBS and infected with 10 genome equivalents per 1 cell. JJhan and SupT1 T cells were grown as already described [15].

Transfection of U94 expression plasmid was performed by nucleofection (Amaxa, Lonza), upon standard conditions, as described [27]. Control cells received the same amount of empty vector alone. Efficiency of transfection, determined in parallel samples by transfection with pmax-GFP plasmid was approximately 70% in all experiments.

Cell free virus inocula and UV-inactivated viral preparations were obtained as described [15]: HHV-6 variant A (strain U1102) was grown and analyzed in the JJhan cell line [15]; HHV-6 variant B (strain Z29) and HHV-7 (strain CZ) [51] were grown in the Sup-T1 cell line [52].

*In vitro* virus infection was performed in Nthy-ori3-1 cells seeded at optimal density as previously described [15], [27].

### DNA and RNA analyses

DNA and RNA were isolated from clinical samples and Nthy-ori3-1 cells as described [27]. All RNA preparations were devoid of DNA, as assured by multiple DNase digestions and lack of amplification in PCR reactions where retrotranscription (RT) had been omitted [14]. HHV-6 DNA presence and load were analyzed by PCR and real time quantitative (qPCR) specific for the U94 and U42 genes [27], and samples were considered positive when 1 µg of cell DNA harbored more than 100 copies of viral DNA [27]. Amplification of the house-keeping human RNase P gene was used as a control. All clinical samples were analyzed in a randomized and blinded fashion. In addition, 15/28 control and 21/34 HT FNAs, when there was enough material to repeat the analysis, were tested again in a randomized and blinded fashion at a distant time from the first analyses.

HHV-6 variant A or B identification was obtained by restriction enzyme digestion with HindIII enzyme of the U31 nested PCR amplification product, as reported previously [26]. Digestion products were then visualized on ethidium bromide stained agarose gel after electrophoresis migration.

Virus transcription was assessed by PCR or qPCR after retrotranscription (RT-PCR, RT-qPCR), determining the presence of lytic (U42, U22) or latent (U94 in the absence of U42) mRNAs, as previously reported [27]. The sensitivity of the used PCRs was similar for all genes, detecting as few as 100 copies of target sequence.

Cell fractions derived by immunomagnetic separation of FNAs were characterized by RT-PCR specific for leukocytes transcripts (respectively CD45, CD3), using serial dilutions of cDNA template, corresponding to amounts of total extracted RNA ranging from 100 ng to 1 pg. Primers and PCR conditions for CD3 and CD45 were previously reported [53], [54], and amplification reactions were carried out for 30 cycles. In each assay the cDNAs obtained from JJhan T cells or Nthy-ori3-1 thyroid cells were also included as positive and negative controls respectively. Amplification of the house-keeping β-actin gene was used as a control.

### Immunofluorescence assay

Immunofluorescence for HHV-6 antigen expression was performed with a mouse monoclonal antibodies (mAb) directed against glycoprotein gp116 (late antigen) of HHV-6 A and B (ABI, Columbia, MD, USA), as previously described [15].

### HLA class I and class II expression

Expression of HLA class I and II (DR) antigens was investigated in Nthy-ori3-1 cells infected with HHV-6 or HHV-7, transfected with U94 expression plasmid or treated with IFN-γ (10 U/ml) [15]. Staining was performed with anti-HLA-I (W6/32, IgG2a, FITC), anti-HLA-DR mAb (L243, IgG2a, PECy5.5) (Caltag Laboratories, CA, USA), and isotypic controls (Exbio, Praha, Czech Republic). The analysis was carried out with a FACSCount cytometer and the CellQuest software (Becton Dickinson, San Jose, CA, USA). Results were expressed as MFI (mean fluorescence intensity).

### Degranulation and apoptosis assays

The CD107a mobilization assay was performed using infected or non-infected Nthy-ori3-1 cells as target cells and PBMC from controls or HT patients as effector cells, with an effector∶target ratio of 2∶1. K562 cells were used as positive control of NK activation. Degranulation was assessed in triplicate after 4 hours of co-culture by staining with PE-Cy5-conjugated anti-CD107a mAb (e-Bioscience, Frankfurt, DE) and gating on CD3−/CD56+ NK cells [55], [56]. Induction of apoptosis was evaluated by flow cytometry after staining with FITC-labeled Annexin-V (Bender MedSystem, Vienna, AU). Cell viability was assessed by Propidium Iodide staining. Results were expressed as percentage of gated cells.

### Dual color ELISPOT assay

IFN-γ and IL-2 secretion by HHV6-specific CD4+ and CD8+ T cells was quantified using a dual color ELISPOT assay in 20 HT patients and 20 controls. Assays were performed using either total virus lysate or purified recombinant HHV-6 U94 protein as antigens. Briefly, an aliquot of purified HHV-6A containing 10^10^ virus genomes/ml was lysed with 0.25% Triton X-100 followed by sonication. Stock solutions were used at a 1∶1,000 dilution in the final assay. HHV-6 recombinant U94 protein, obtained as described [15], was used at 2 µg/ml. CD4+ and CD8+T cells were isolated from PBMCs by immunomagnetic separation (Miltenyi Biotec, Calderara di Reno, Italy). Aliquots of 5×10^4^ CD8+ T cells, seeded in anti-IFNγ and -IL-2 antibody coated wells, were stimulated with the antigens. Mock lysates, obtained from uninfected JJhan cells, or Tetanus toxoid (TT, 5 µg/ml, Calbiochem, San Diego, CA) were also used, respectively as negative and positive controls. Results are expressed as spot forming cells (SFC) per 5×10^4^ CD4+ or CD8+ T lymphocytes.

### Statistical analysis

Statistical significance of results was analyzed by independent Student t-test.
